# Age does *not* count: resilience of quantity processing in healthy ageing

**DOI:** 10.3389/fpsyg.2013.00865

**Published:** 2013-12-10

**Authors:** Anna Lambrechts, Vyacheslav Karolis, Sara Garcia, Jennifer Obende, Marinella Cappelletti

**Affiliations:** ^1^Autism Research Group, Department of Psychology, City University LondonLondon, UK; ^2^Institute of Cognitive Neuroscience, University College LondonLondon, UK; ^3^Institute of Ophthalmology, University College LondonLondon, UK; ^4^Psychology Department, University College LondonLondon, UK

**Keywords:** quantity processing, time, space, number, ageing, magnitude system

## Abstract

Quantity skills have been extensively studied in terms of their development and pathological decline. Recently, numerosity discrimination (i.e., how many items are in a set) has been shown to be resilient to healthy ageing despite relying on inhibitory skills, but whether processing continuous quantities such as time and space is equally well-maintained in ageing participants is not known. Life-long exposure to quantity-related problems may progressively refine proficiency in quantity tasks, or alternatively quantity skills may decline with age. In addition, is not known whether the tight relationship between quantity dimensions typically shown in their interactions is preserved in ageing. To address these questions, two experimental paradigms were used in 38 younger and 32 older healthy adults who showed typical age-related decline in attention, executive function and memory tasks. In both groups we first assessed time and space discrimination independently using a two-choice task (i.e., “Which of two horizontal lines is longer in duration or extension?”), and found that time and space processing were equally accurate in younger and older participants. In a second paradigm, we assessed the relation between different quantity dimensions which were presented as a dynamic pattern of dots independently changing in duration, spatial extension and numerosity. Younger and older participants again showed a similar profile of interaction between number, cumulative area and duration, although older adults showed a greater sensitivity to task-irrelevant information than younger adults in the cumulative area task but lower sensitivity in the duration task. Continuous quantity processing seems therefore resilient to ageing similar to numerosity and to other non-quantity skills like vocabulary or implicit memory; however, ageing might differentially affect different quantity dimensions.

## Introduction

A central part of our everyday life involves judging quantities, for example which queue at the supermarket has fewer people, or if a parking space is wide enough for our car, or if there is sufficient time to pop to a café before our next meeting (Lemaire and Lecacheur, 2007; Gandini et al., 2008, 2009). Collectively these judgments provide rough magnitude estimates in the form of number, spatial extension or temporal duration (Walsh, 2003; Gandini et al., 2009; Bueti and Walsh, 2009; Bonn and Cantlon, 2012; Cantlon, 2012).

A large body of research has recently investigated the development of numerical, spatial and temporal estimations in humans and primates and their impairment in the lesioned brain. For instance, these studies have shown that different magnitude dimensions have parallel patterns of performance in animals (Meck and Church, 1983; Breukelaar and Dalrymple-Alford, 1998; Meck, 2005; Beran, 2007; Merritt et al., 2010), and similar rates of development in humans (Brannon et al., 2006, 2007; Van Marle and Wynn, 2006; Feigenson, 2007; Droit-volet et al., 2008; Reynvoet et al., 2009). It has also been shown that there are associations (Basso et al., 1996; Zorzi et al., 2002) and dissociations between dimensions in the lesioned brain (Doricchi et al., 2005; Cappelletti et al., 2009, 2011). This evidence has supported the idea that magnitude dimensions are mapped onto an abstract analogue scale (Walsh, 2003; Bueti and Walsh, 2009; Gallistel, 2011) such that from early in development individuals apply associative mappings “more A, more B” across different magnitude dimensions (Lourenco and Longo, 2010).

### Magnitude processing in healthy ageing

Some research has investigated math and numerosity processing in older age, but processing of continuous quantities such as space and time in ageing is less known. Existing studies on number have focused on elderly's mathematical abilities (Halberda et al., 2012; Duverne and Lemaire, 2005; Dormal et al., 2012), and recently on more foundational skills such as numerosity discrimination (Halberda et al., 2012; Cappelletti et al., in press). These studies concurred to show that although older participants can learn new ways to solve arithmetical problems, they show a smaller repertoire of strategies and are less efficient than younger participants in selecting among them (e.g., Duverne and Lemaire, 2005; Lemaire and Arnaud, 2008), or that they do not equally engage the same brain regions as younger participants when performing arithmetical tasks (El Yagoubi et al., 2005). Moreover, Cappelletti et al. (in press) found that numerosity discrimination is resilient to ageing although it is influenced by the decline of inhibitory processes supporting number performance. In comparison to number, time and space processing have been much less investigated in older adults (OAs). Some evidence in the temporal domain indicates that OAs demonstrate diminished accuracy but intact sensitivity in duration judgments (Baudouin et al., 2006; Block et al., 1998; Lustig and Meck, 2011). For instance, OAs report larger estimates but reproduce shorter durations relative to younger adults (YAs) (Block et al., 1998). However, these group differences might reflect age-related declining of skills required for temporal judgments, like working memory storage and executive functions, in which case they would not be suggestive of a pure deficit in temporal processing in ageing. In the domain of spatial processing, differences in speed (Birren and Botwinick, 1955) but not in accuracy (Verrillo, 1981; but see Sara and Faubert, 2000) have been reported between YAs and OAs in size discrimination tasks.

### Relations between magnitude dimensions from infancy to older age

One way to probe the integrity of magnitude processing lays in examining interaction effects between magnitude dimensions, and interactions are at the basis of the idea that different dimensions are mapped on an analogue scale, and that magnitude processing skills rely on common resources (Gallistel, 1989, 2011; Gallistel and Gelman, 2000; Walsh, 2003; Bueti and Walsh, 2009; Cantlon, 2012). Interaction studies have shown that judgments on a target dimension are sensitive to information from concurrent task-irrelevant magnitude dimensions. Such studies usually examine the influence of one dimension on the other, unilaterally (effect of A on B) or bilaterally (effect of A on B and effect of B on A). For instance, duration has been recurrently found to be sensitive to task-irrelevant numerical information (both symbolic, like Arabic figures and non-symbolic, like number of dots) following a “more A, more B” pattern: the larger the number, the longer the duration is perceived (Droit-Volet et al., 2003; Dormal et al., 2006; Xuan et al., 2007, 2009; Oliveri et al., 2008; Vicario et al., 2008; Dormal and Pesenti, 2013). Duration processing has also been shown to be unilaterally sensitive to spatial interaction. The longer the length or size of a stimulus (physical or implicit), the longer its duration is perceived (Xuan et al., 2007; Casasanto and Boroditsky, 2008; Bottini and Casasanto, 2010; Casasanto et al., 2010; Dormal and Pesenti, 2013). In contrast, studies report that duration does not influence numerical judgments (Droit-Volet et al., 2003; Dormal et al., 2006; Dormal and Pesenti, 2013). Similarly, although the classic tau effect (Helson and King, 1931) is an example of the influence of duration on spatial judgment, this finding has often not been replicated, leading to the suggestion that duration does not influence spatial judgments (Casasanto and Boroditsky, 2008; Bottini and Casasanto, 2010; Dormal and Pesenti, 2013). An exception to this pattern of results has been shown in a few recent studies in which interactions between number and duration have been reported to be bidirectional (Arend et al, under review; Javadi and Aichelburg, 2012, 2013). Likewise, spatial and numerical dimensions have been shown to interfere with each other bidirectionally (space affects number perception and number affects space perception), although not always symmetrically (interactions can be stronger in one direction than the other). Most studies report again a “more A, more B” pattern: the larger the numerical (symbolic or non symbolic) magnitude, the longer the length of a line is perceived (Dormal and Pesenti, 2007, 2013; De Hevia et al., 2008; De Hevia and Spelke, 2010); reciprocally, the longer the size, the larger the number is perceived (Dormal and Pesenti, 2007, 2013; although see Shuman and Spelke, 2006 and Tokita and Ishiguchi, 2011).

Interactions between dimensions have been proposed to be the side product of an automatic mapping of number, space and time on an analogue magnitude (Cantlon, 2012; Dormal and Pesenti, 2013). Alternatively interactions could be the manifestation of a statistical relationship between numerical, spatial and temporal information that we extrapolate to refine magnitude estimations (Cantlon, 2012): if we observe consistently that longer distance take a longer time and a larger number of steps to walk, we can correct our estimate of the length path by estimating the duration of the trip and the number of steps we made. In both cases, interactions reflect a tight relationship between the processing of different magnitude dimensions. To the best of our knowledge no research has yet assessed whether OAs present the same pattern of interactions at those observed in younger individuals.

### Objectives of the current studies

Here we investigated time and space processing first independently, and then in combination in young and ageing participants. In a first experiment we assessed spatial and temporal processing in OAs using a well-established psychophysics paradigm. In addition, using dedicated and well-known neuropsychological measures, we investigated the integrity of older participants' arithmetical, memory, attention and executive processes which might reflect or contribute to any age-related difference in quantity skills. We reasoned that if performance in the spatial and temporal processing tasks did not differ between older and younger participants, this may be suggestive of maintained temporal and spatial judgments in ageing, or of compensatory mechanisms in OAs to palliate to the general cognitive decline associated with ageing. Performance in tasks assessing auxiliary processes (memory, attention, executive processes) provided us with a measure of cognitive decline, allowing us to evaluate its relation to performance in time and space discrimination. In contrast, age-related differences in time and space discrimination may reflect impairments specific to a single dimension, or impairments of the whole quantity system. In a second experiment we examined the relationships between different dimensions in ageing and probed whether magnitude dimensions interfere with each other in a similar fashion in OAs and in YAs. We reasoned that a similar pattern of interactions in the two groups, albeit different in amplitude (e.g., stronger or weaker in the OAs group), may indicate that the magnitude system is robust and resilient to ageing. Alternatively, if magnitude dimensions are differently affected by ageing, the pattern of interactions itself (i.e., directionality of the interactions) is expected to differ from YAs' without necessarily showing weaker or stronger interactions.

## Participants

A total of 70 right-handed neurologically healthy participants with normal or corrected-to-normal vision gave written consent and were paid to participate in our study which was approved by the local research Ethics Committee. Participants were selected from the UCL Institute of Cognitive Neuroscience database based on their age. Forty-five participants took part in Study 1: 24 were young participants with a mean age of 24.8 years (*SD* = 3.64; age range 20–35; 9 males); 21 were older participants with a mean age of 65 years (*SD* = 4.8; age range 59–74; 10 males). Thirty participants took part in Study 2: 16 were young participants with a mean age of 25.0 years (*SD* = 4.4; age range: 20–37; 9 males); 14 were older participants with a mean age of 66.9 years (*SD* = 3.4; age range: 63–73; 6 males). Two young and three older participants took part in both studies.

## Study 1

We first examined whether processing the continuous dimensions of time and space may be affected by ageing. We used an established experimental paradigm previously employed to probe continuous quantity processing in young healthy participants and neurological patients (Cappelletti et al., 2009, 2011), whereby in different blocks participants were asked to discriminate duration or spatial extension (length) on one-dimensional stimuli (horizontal lines).

### Methods

#### Background tasks

Participants in both groups were assessed with standard tests of intelligence (National Adult Reading Test, Nelson and Willison, 1991) and vocabulary (vocabulary subtest of the WAIS-R, Wechsler, 1995). They were also tested on the Attention Network Test (Fan et al., 2002), the color Stroop task (Stroop, 1935) and the number Stroop task (Henik and Tzelgov, 1982) to assess attentional and inhibitory functions (see description of the tasks below); the “Doors and People” test (Baddeley et al., 1994) as well as the digit span and the spatial span (Wechsler, 1995; see description of the tasks below) were administered to test memory performance. In addition, OAs were given the Mini Mental State Examination (Folstein et al., 1975) to screen for cognitive impairment.

*The Attention Network Test* (ANT, Fan et al., 2002) examines executive and inhibitory processes by asking participants to attend to one target while ignoring others (Posner et al., 1980). Three aspects of performance are measured: alertness, orienting, and conflict. The version used here combined a cueing task and a flanker task (Eriksen and Eriksen, 1974): participants responded to cued or un-cued central targets while ignoring flanking distractors. The stimuli consisted of a target arrow flanked by two arrows on either side, which could point to the same direction as the target arrow (congruent condition, e.g.,→→→→→) or to the opposite direction (incongruent condition, e.g.,→→←→→). Following Fan et al. (2002), each arrow was presented at 0.55° of visual angle and separated from the adjacent arrows by 0.06° of visual angle. The stimuli (central arrow and flankers) measured 3.08° of visual angle in total. Participants were instructed to attend to the middle arrow and to decide whether it was pointing to the left or to the right. Each trial started with a central fixation cross which was presented for a random duration between 400 and 1600 ms, followed by either a 100 ms warning asterisk cue (cued trials) or by a longer fixation (un-cued trials), and by a second 400 ms fixation period after which the target and the flankers appeared simultaneously and centrally at 1.06° of visual angle either above or below the fixation point. The cue was always valid and could either appear centrally, i.e., in a spatially neutral condition or precede the target and flankers in the same position above or below the fixation point, i.e., in a spatially-orienting condition. The target and flankers remained on the screen until the participant responded or for a maximum of 1700 ms. The next trial began immediately after a response was made. A total of 288 trials were presented in 3 blocks of 96 trials each. Responses were made by pressing a left-hand key (or right-hand key) if the central arrow pointed left (or right) as quickly as possible.

*The color Stroop task* (Stroop, 1935) provides a standard measure of participants' ability to inhibit task-irrelevant information. Participants are instructed to report as quickly as possible the color of the font in which words are displayed while ignoring their meaning. In each trial, participants saw a centrally presented 500 ms fixation cross, followed by a word stimulus which stayed on the screen until the participant made a response or for a maximum of 4000 ms. The following trial started immediately. The task consisted of a total of 60 trials. Stimuli were either the words “RED” and “BLUE” or a string of “XXX.” The color of the font was red or blue, resulting in congruent (e.g., the word RED appearing in red), incongruent (e.g., the word RED appearing in blue) and neutral conditions (e.g., XXX appearing in red). There were 20 trials in each condition. Responses were given by pressing the left or right arrow keys for blue or red color of the font, respectively.

*The number Stroop task* (Henik and Tzelgov, 1982) assesses the automatic processing of numbers as well as inhibitory processes using stimuli that contain congruent and incongruent information. In two separate tasks, participants viewed a total of 336 pairs of 1–9 Arabic numbers (168 per block) that could vary in numerical magnitude (e.g., 3 vs. 2) or physical size (e.g., 3 vs. 2). There were three types of stimuli (36 trials for each type): a pair in which the digit larger in magnitude was also larger in size was a congruent stimulus; a pair in which digits did not differ in one of the two dimensions was a neutral stimulus; a pair in which the digit larger in magnitude was smaller in size was an incongruent stimulus. Each number stimulus could be paired to itself, therefore consisting of a neutral stimulus for the physical size condition (e.g., 2 vs. 2), or to another number stimulus which could be between 1 and 4 units apart. Moreover, the two number stimuli could be of the same physical size, therefore consisting of the neutral stimulus for the numerical magnitude condition, or they could vary along two levels of physical size, as stimuli could appear in a vertical visual angle of 0.7 or 0.9° centered along the horizontal line of the computer screen to the left or the right of the fixation cross. Participants indicated on which side was the larger number in either numerical magnitude or physical size by pressing either the left or the right arrow. A trial started with a 500 ms fixation cross, followed by the number stimuli until the participant made an answer or for a maximum of 4000 ms. After this, the following trial started immediately. For each task (number or physical size), accuracy and response times were recorded. This experimental paradigm commonly shows a “facilitation effect,” i.e., participants are faster to respond to congruent stimuli (e.g., 3 vs. 2) relative to neutral stimuli (e.g., 3 vs. 3 for physical comparisons or 3 vs. 2 for numerical comparisons), they are slower to respond to incongruent stimuli (e.g., 3 vs. 2) relative to neutral stimuli (Henik and Tzelgov, 1982).

*The “Doors and People” Recognition test* (“Doors” stimuli only) was used to assess visual memory (Baddeley et al., 1994). Participants were asked to memorise the images of two sets consisting of 12 pictures of doors, which were presented sequentially for 3 s each. Immediately after, participants were asked to indicate with no time pressure which image they had previously seen amongst a choice of 4 images, three of which were new. In the first set of pictures, new and old door stimuli differed on general appearance; in the second set, old and new door stimuli differed in finer details (more difficult).

*The digit span task* (Wechsler, 1995) was used to assess verbal working memory. Here participants were instructed to repeat increasingly longer sequences of number stimuli presented verbally. The sequences increased in length by one item until a participant could not repeat two sequences of the same length without making an error. In a first block, the sequences had to be repeated in the forward order; in a second block, they were repeated in the reversed order.

*Spatial span* assessed spatial working memory using the “Corsi” task (Wechsler, 1995). Participants observed the experimenter touching a series of blocks on a horizontal board in a given sequence. Participants were then instructed to repeat the same steps in each sequence. Sequences increased progressively in length by one unit and were repeated in the forward order only.

#### Experimental tasks: continuous quantity processing

Stimulus presentation and data collection were controlled using the Cogent Graphics toolbox (http://www.vislab.ucl.ac.uk/Cogent) and Matlab 7.0 software on a Sony-Vaio laptop computer. The dimensions of the display, as rendered on the built-in liquid-crystal screen, were 33.8 cm horizontal by 27 cm vertical. The display had a resolution of 1280 × 1024 pixels and was refreshed at a frequency of 60 Hz. A chin-rest was used to stabilize head position of the participants and the viewing distance from the monitor was 50 cm. During all testing sessions participants sat in a quiet room facing the computer screen under normal room lighting.

**Figure 1:**
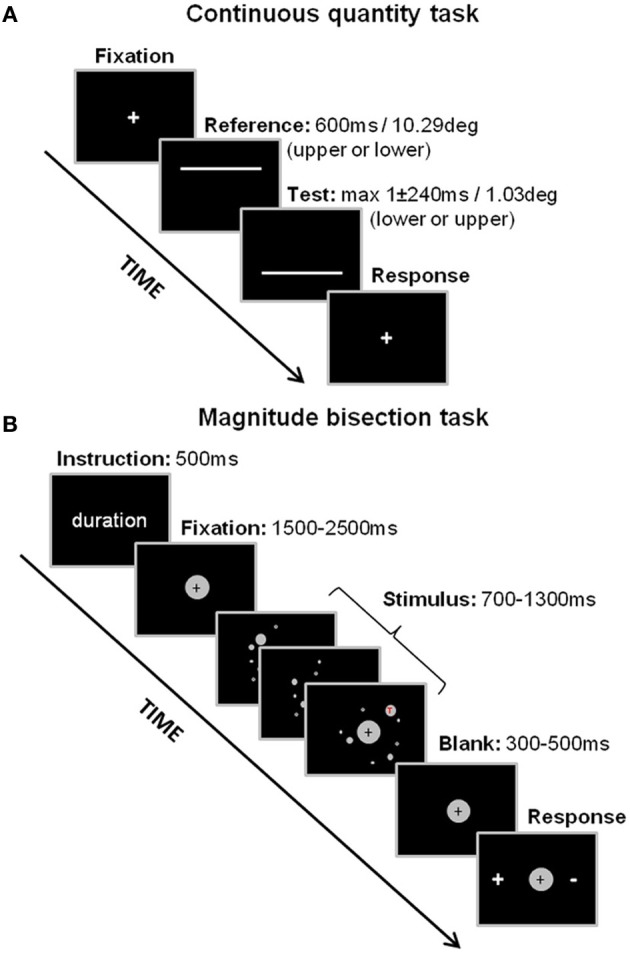
**Experimental designs for the continuous quantity tasks (A) and the magnitude bisection tasks (B)**. In the continuous quantity tasks, participants had to compare the length or the duration of two lines presented sequentially on the screen. In the magnitude bisection tasks, participants had to decide whether the number of dots, cumulative area covered by dots or duration of the display was closer to a small/short or a large/long standard.

***Stimuli***. Stimuli were two horizontal white lines (thickness 0.153°) centered on the vertical meridian on a black background. The lines were presented sequentially in a two-interval discrimination paradigm, one line 5.07° above the horizontal meridian and the other 5.07° below (see Figure 1A). The first line stimulus in the two-interval sequence (the “Reference”) always had a length of 10.06° and a duration of 600 ms. The second line (the “Test”) could vary according to a Method of Constant Stimulip either in length or duration, depending on the dimension to be judged (the irrelevant dimension always matched the Reference). For each dimension the ratio between the smaller and the larger stimulus could vary unpredictably over five levels (steps of 0.201° for length and 40 ms for time) with equal frequency: ratio of 1.02, 1.04, 1.06, 1.08, and 1.10 for length and ratio of 1.067, 1.133, 1.20, 1.267, 1.333 for time, selected from previous pilot studies. There were 5 blocks of 40 observations for each level of the test stimulus (total of 200 observations for each task). The length and duration discrimination tasks were run independently from each other in counterbalanced order across participants to avoid order effects (see Figure 1A).

***Design***. Each trial began with a centrally displayed fixation point (diameter 0.153°), which remained visible until a key-press from the participant. The Reference (or the Test) line was then immediately displayed above (or below) the fixation point followed by the Test (or the Reference) line below (or above) the fixation, and an inter-stimulus-interval of 100ms. The screen then remained blank until a response from the subject, followed by the central fixation point which stayed on the screen until the participant pressed the space bar; the next trial followed immediately (see Figure 1A).

***Procedure***. In each task, participants were instructed to make un-speeded responses by pressing either the “up” or “down” cursor-arrow keys of the computer keyboard to indicate the vertical position of the test line which appeared the longest either in duration or in spatial extent. Correct answers were equally assigned to the “up” or “down” keys in each task.

### Data analysis

For both *color and number Stroop tasks* we calculated the difference in RT between congruent and incongruent trials, considered to be a standard measure of participants' ability to inhibit task-irrelevant information (Stroop, 1935). In further analyses we refer to this index as the Stroop effect.

In the *duration and length discrimination tasks*, each participant's response distributions were used to estimate the precision of the underlying magnitude representation, expressed as the Weber fraction (*wf*). The magnitude representations were assumed to be Gaussians with standard deviations linearly related to their means. The *wf* determines the variation of the standard deviation of the Gaussian random variables in each magnitude. The estimates of *wf* were obtained by fitting cumulative Gaussian function with log-transformed test magnitude as a predictor to the proportions of correct responses for each test level. The data were fitted using maximum likelihood criterion. The fitting function had the standard deviation as the only free parameter. The mean of cumulative Gaussian was fixed at the magnitude of Reference along relevant dimension. The standard deviations of the fitted functions were then divided by the square root of 2 to obtain the estimates of individual *wf*. A larger *wf* implies a larger overlap between two magnitude representations leading to a lower discriminability and a higher rate of incorrect responses. Therefore, a large *wf* indicates a worse performance in the task.

In the *ANT* three indexes of performance were measured based on how response times of correct answers are influenced by alerting cues, spatial cues, and flankers: alertness (cued vs. un-cued trials), orienting (central cue vs. spatial cue), and conflict (congruent vs. incongruent trials averaged across cued and un-cued, and central vs. spatial cue).

In all tasks, data were analyzed using ANOVA and *t*-tests with a *p*-value <0.05 considered significant for all statistical analyses. For the standardized tasks (with the exclusion of IQ, digit span, spatial span, Doors and People task and vocabulary) non-parametric tests were used (Mann–Whitney *U*-test).

In Study 1, a total of 4.84 and 4.86% of the data were missing in the older and in the younger group, respectively. The data sets were completed using expectation-maximization protocol implemented in the SPSS package. Only one data point was missing in the time discrimination in the older group and none in the young group. There were no missing data points for the space discrimination task.

### Results

#### Background tasks

There was a significant group difference in IQ and vocabulary scores, with older participants outperforming the young [*t*_(43)_ = 3.83, *p* < 0.001, *p* = 0.005, and *z*-score approximation = 2.78, respectively], consistent with results reported in previous studies (Hedden and Gabrieli, 2004). Performance on the Mini Mental State Examination (Folstein et al., 1975) showed no signs of cognitive deterioration in older participants (see Table 1).

**Table 1:** **Demographic data and descriptive statistics for the younger adult (left) and older adult (right) groups in Study 1**.

**Task/information**	**Younger participants (*N* = 24)**	**Older participants (*N* = 21)**
**A. DEMOGRAPHIC INFORMATION**		
Age	24.8 years (*SD* = 3.6)	65.0 years (*SD* = 4.8)
Gender	9 males	10 males
**B. BACKGROUND**		
Full IQ (NART^a^)	114.3 (*SD* = 11.22)	125.7 (*SD* = 8.4)
Mini Mental State Examination^b^	nt	Median = 30 (min = 28)
Vocabulary^c^	Median = 50 (IQR = 7)	Median = 57 (IQR = 7.5)
**C. ATTENTION AND EXECUTIVE FUNCTIONS AND MEMORY**		
Attention network test (ANT)^d^		
Orienting	37.3 ms (*SD* = 18.5)	54.0. ms (*SD* = 34.4)
Alerting	24.9 ms (*SD* = 15.9)	3.5 ms (*SD* = 41.8)
Conflict (incongruent-congruent)	85.9 ms (*SD* = 20.5)	116.3 ms (*SD* = 36.1)
Word Stroop task (Stroop effect)^e^	21.5 ms (*SD* = 31.1)	111.5 ms (*SD* = 89.9)
Number Stroop task (Stroop effect)^f^		
Numerical comparison	68.3 ms (*SD* = 36.6)	110.3 ms (*SD* = 54.8)
Physical comparison	51.1 ms (*SD* = 35.0)	69.1 ms (*SD* = 39.4)
Visual memory (Door recognition)^g^	Median = 22 (IQR = 2)	Median = 20 (IQR = 4.5)
Verbal memory (Digit span)^a^	Median = 22 (IQR = 8)	Median = 22 (IQR = 6)
Spatial memory (Spatial span)^a^	Median = 10 (IQR = 6)	Median = 9 (IQR = 4.5)
**D. CONTINUOUS QUANTITY PROCESSING**		
Time discrimination (*wf*)	0.32 (95% CI: 0.06–1.65)	0.30 (95% CI: 0.097–0.92)
Space discrimination (*wf*)	0.043 (95% CI: 0.022–0.084)	0.039 (95% CI: 0.012–0.127)

a Nelson and Willison, 1991;

b Folstein et al., 1975; max score:30

c Wechsler, 1995;

d Fan et al., 2002;

e Stroop, 1935;

f Henik and Tzelgov, 1982;

g Baddeley et al., 1994;

*SD, Standard deviation; IQR, Interquartile Range; CI, Confidence Interval; nt, not tested; ms, milliseconds; wf, Weber Fraction*.

Older participants performed worse than young in tests assessing attention (ANT); specifically they were significantly slower at orienting [*t*_(43)_ = 2.07, *p* < 0.044] and alerting attention following visual cues [*t*_(43)_ = 2.33, *p* = 0.025]. Older participants were also worse than younger participants in processing stimuli containing conflicting information [*t*_(43)_ = 3.52, *p* = 0.001].

Executive functions measured in the color Stroop task also indicated group differences. Both groups showed reliable Stroop effect [YA: *t*_(23)_ = 3.38 *p* < 0.005; OA: *t*_(20)_ = 5.68, *p* < 0.001] but this was stronger in the older group (YA mean RT difference: 21.5 ms, *SD* = 31.1; OA mean RT difference: 111.5 ms, *SD* = 89.9, *t*_(43)_ = 4.61, *p* < 0.001), indicating a difficulty for OAs to inhibit task-irrelevant information.

In the number Stroop task, an ANOVA with task (number and physical size comparison) and group (older and younger) factors showed a main effect of task [*F*_(1, 43)_ = 9.78, *p* = 0.003, η^2^_*p*_ = 0.23] and of group [*F*_(1, 43)_ = 13.14, *p* = 0.001, η^2^_*p*_ = 0.31] but no significant interaction. Across the groups, the effect of physical size on numerical comparison was stronger than vice versa (mean Stroop effect for size-relevant task: 59.5 ms, *SD* = 37.8; mean Stroop effect for number-relevant task: 87.93 ms, *SD* = 50.13). Across tasks, there was a greater Stroop effect in the older than in the younger group (mean RT difference for YA: 59.7 ms, *SD* = 21.5; mean RT difference for OA: 89.72 ms, *SD* = 33.44), consistent with the result of the color Stroop task.

Visual memory function measured with the “Doors and People” task showed a significant group difference indicating a better performance in the younger group (younger vs. older: *p* < 0.001, *z*-score approximation = 3.99). A marginally significant group difference was observed in the task measuring spatial span (YA vs. OA: *p* < 0.074, *z* = 1.79) but not digit span (YA vs. OA: *p* < 0.79, *z* = 0.26).

#### Experimental tasks: continuous quantity processing

**Figure 2:**
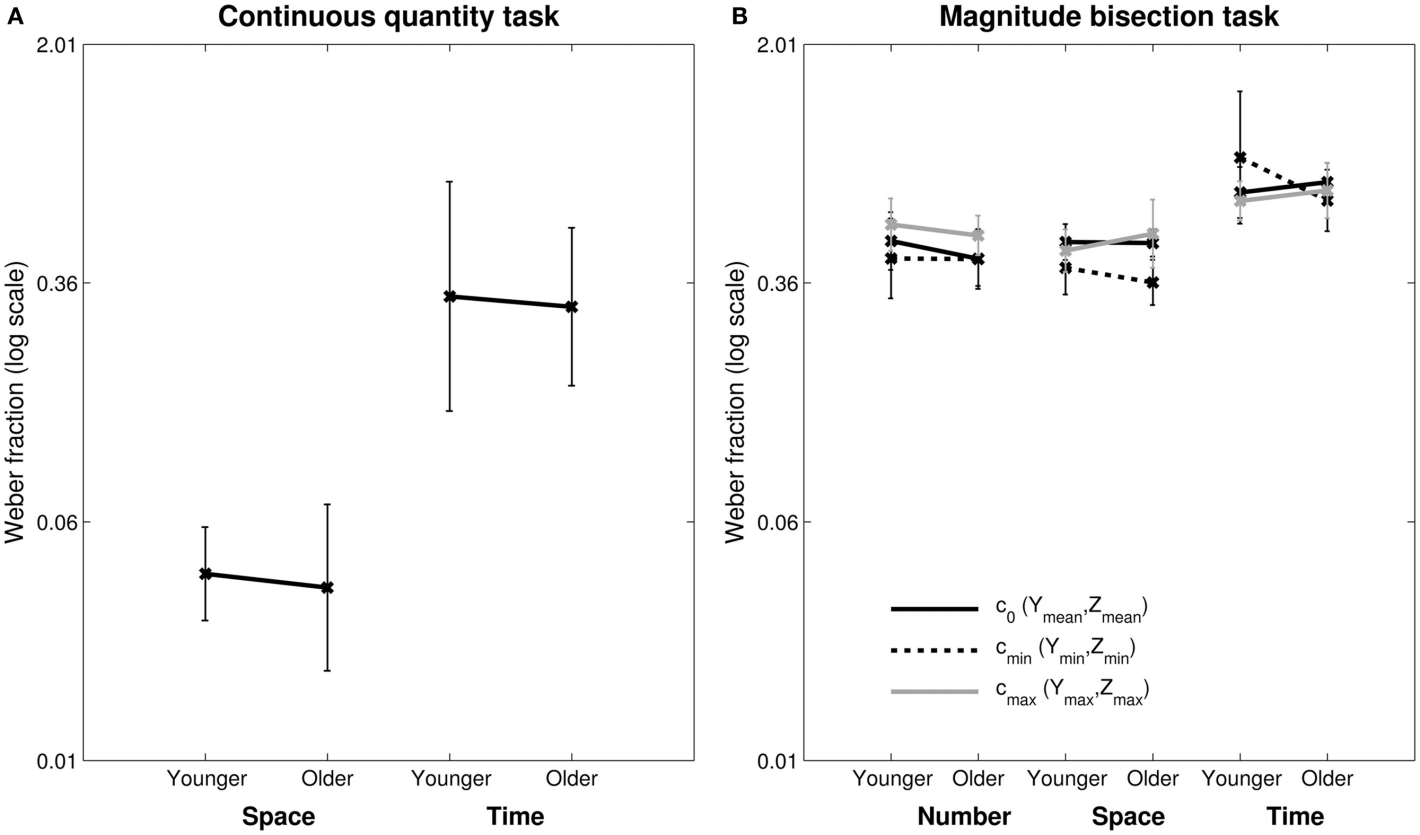
**Weber Fractions (*wf*) for the continuous quantity tasks (A) and the magnitude bisection tasks (B)**. In the magnitude bisection tasks *wf* are presented in the three experimental conditions *c*_0_ (*Y*_mean_, *Z*_mean_), *c*_min_ (*Y*_min_, *Z*_min_) and *c*_max_ (*Y*_max_, *Z*_max_). Weber Fractions are a measure of sensitivity in discrimination tasks. Error bars show standard deviations.

We first tested whether there was any group difference in any of the continuous quantity tasks. An ANOVA with the log *wf* of duration and length tasks as within-subject factor and group (younger and older) as between-subject factor showed only a significant main effect of task [*F*_(1, 43)_ = 356.42, *p* < 0.001, η^2^_*p*_ = 0.89, see Figure 2, left panel]. Specifically *wf* was higher in the duration task than in the length task (0.31 and 0.041, respectively), indicating that participants across groups demonstrated higher precision to judge length than duration. Further analyses specific for each task show no group difference [Space: *t*_(43)_ = 0.7, *ns*; time: *t*_(53)_ = 0.34, *ns*].

We also used a regression analysis to investigate whether, within the older group, age may predict performance in space and time processing. An analysis based on regressing the log *wf* on participants' age showed no negative effect of age on performance (space: *t* = 0.86, *p* = 0.40, *R*^2^_adj_ = 0.0; time: *t* = 1.57, *p* = 0.13, *R*^2^_adj_ = 0.07; where a negative *t*-value implies decline with age).

There was a significant correlation between *wf* of length and duration tasks in the older group, *r* = 0.60, *p* = 0.004, but not in the younger group (*r* = 0.16, *p* = 0.46). However, the comparison of correlations using Fischer's Z transformation failed to show a significant group difference (*z* = 1.64, *p* = 0.10).

Next, we examined whether both within and across groups continuous quantity processing correlated with other cognitive abilities, especially the inhibitory ones. There was no correlation with measures of IQ, vocabulary, attention (orienting, alerting and conflict separately), spatial, visual and verbal memory across groups. However, in the older group better performance in duration and length discrimination negatively correlated with the Stroop effect measured in the color Stroop task (Time: *r* = 0.66, *p* = 0.001; Space: *r* = 0.45, *p* = 0.036). In other words, older participants who could better resolve conflict were also better at discriminating continuous quantities. Length discrimination also negatively correlated with a measure of conflict resolution in ANT task, (*r* = 0.50, *p* = 0.021), but not with orienting and alerting. No correlation with the Stroop effect in the number Stroop task was observed.

These results suggest that time and space discrimination were maintained in ageing participants, who showed otherwise typical signs of healthy cognitive ageing in memory, attention and executive functions. However, in the OAs group only, better performance in time and space discrimination tasks was related to their better ability to resolve conflict. This could indicate that OAs rely more on inhibitory processes than YAs when discriminating length and duration, either to suppress the tendency to answer the second of two stimuli (presentation-order effect: Hellström, 1985; Masin and Fanton, 1989), or to solve the conflict between two competing choices. In addition, whereas OAs' performance in duration and length discrimination correlated with each other, YAs' performance did not relate to each other or to any of the cognitive functioning measures that we collected. This is consistent with a recent study looking at the behavioral and anatomical links between number and space (Cappelletti et al., in press), but it contrasts with the finding that performance in number and cumulative area discrimination correlates in young adults (Lourenco et al., 2012). The absence of correlation might indicate a stronger link between space and numerosity processing than between space and time processing. The fact that performance in duration and length discrimination tasks correlates in ageing, however, hints at the possibility that processing of continuous quantities is maintained in ageing but that the link between different dimensions might change with age. We therefore tested a second group of participants with a novel experimental design previously used to probe interactions between magnitude dimensions (Lambrechts et al., in press). We reasoned that weaker or stronger interactions between dimensions in OAs may indicate a smaller or larger reliance on common processes for magnitude discrimination, respectively. Alternatively, a pattern of interactions between dimensions which is altogether different from YAs' may suggest that age-related changes might be dimension-specific.

## Study 2

Here we specifically examined whether known interactions between magnitude dimensions (time, space and numerosity) are maintained in ageing. The stimuli, design and procedure used were adapted from a previous paradigm employed in younger participants (Lambrechts et al., in press). Participants judged the duration, the cumulative area covered by the stimuli, or the number of stimuli (dots) presented in a dynamic display.

### Methods

Stimulus presentation and data collection were controlled using Psychtoolbox 3.0 (Brainard, 1997; Pelli, 1997; Kleiner et al., 2007) and Matlab 7.0 software on a 1024 × 768 pixels monitor screen with a 75 Hz frame rate. Participants were seated ~60 cm away from the display.

#### Background tasks

In addition to the experimental tasks participants were also assessed with a standard test of intelligence (National Adult Reading Test, Nelson and Willison, 1991) and two tests of arithmetic performance (arithmetic subtest in the WAIS-R, Wechsler, 1995 and Graded Difficulty Arithmetic test, Jackson and Warrington, 1986). The latter two were tested in order to evaluate whether a deficit in arithmetic performance may also be present, should duration and cumulative area perception be impaired.

#### Experimental tasks: magnitude bisection tasks

***Stimuli***. Stimuli were dynamic displays of gray dots which appeared and disappeared progressively within a virtual central disk on a black background on the screen (Figure 1B). During one display, dots appeared on the computer monitor in 5–13 steps (to produce a progressive accumulation), 1–8 dots at a time, and they then disappeared progressively after a lifetime of 333–507 ms (all values chosen pseudo-randomly for each trial). Steps during which new dots appeared lasted 40–507 ms. A display was characterized by its duration (time elapsed between the appearance of the first dot and the disappearance of the last one), the cumulative area of its dots, and the total number of dots presented. Duration, cumulative area and number were defined according to 3 experimental conditions (see Design below). Dot stimuli had a radius comprised within 0.45 and 2.84° of visual angle, and could not overlap in space or time. The virtual disk for display had a radius varying pseudo-randomly between 5.7 and 7.7° of visual angle. Dot stimuli were constrained not to appear in an inner disk of radius 0.9° centered on a fixation cross. Luminance of all dots for one trial took one of six values [57, 64, 73, 85, 102, and 128 in the 0(black)-to-255(white) RGB-coded referential] chosen pseudo-randomly. In addition, there was a letter that appeared inside one of the dots, which could be either a red or green, upright or upside-down capital “T” (see Figure 2A). This was used as a control condition to test for participants' general alertness during the task (see below). Participants were asked to discriminate the target (upright red T) from the distracters (upright green T or upside-down red or green T).

***Design***. The experiment combined a bisection task (on magnitude dimensions) and a signal detection paradigm (target/non target). The design for both tasks is summarized in Table 2. For the bisection task, participants were first trained to discriminate between a small (“−”) and a large (“+”) standard in each magnitude dimension (short/long duration, small/large cumulative area, small/big number of dots). During the test phase they were then asked to judge whether the duration, cumulative area or number of dots in each trial was closer to the “−” standard or to the “+” standard. Standards were defined as 0.7 (“−”) and 1.3 (“+”) times a mean value set as *D*_mean_ = 1000 ms, *S*_mean_ = 878 mm^2^, and *N*_mean_ = 28 dots for duration (*D*), cumulative area (*S*), and number (*N*) respectively. These values were chosen to produce similar sensitivity in the three tasks based on Lambrechts et al. (in press). During the test phase each magnitude dimension took 5 possible values defined as 0.7, 0.9, 1, 1.1, and 1.3 times the mean value (hereafter: *X*_0.7_, *X*_0.9_, *X*_mean_, *X*_1.1_, and *X*_1.3_, with dimension *X* being *D*, *S*, or *N*). Three experimental conditions were retained to explore the susceptibility of the target magnitude judgment to irrelevant dimensions (see Figure 2B). In control *condition 0* (*c*_0_), orthogonal dimensions were set to their mean (*Y*_mean_, *Z*_mean_); in *condition 1* (*c*_min_), they were set to their minimal values (0.7 × mean value: *Y*_min_, *Z*_min_) and in *condition 2* (*c*_max_), they were set to their maximal values (1.3 × mean value: *Y*_max_, *Z*_max_).

**Table 2:** **Experimental design for Study 2**.

	**Instruction (target magnitude)**	**Target magnitude value**	**Non-target magnitude value**
Magnitude bisection task	Duration (D)	*X*_min_ = 0.70 *X*_mean_	If target magnitude *D*
	Surface (S)	*X*_0.9_ = 0.90 *X*_mean_	*c*_0_ = [*S*_mean_, *N*_mean_]; *c*_min_ = [*S*_min_, *N*_min_]; *c*_max_ = [*S*_max_, *N*_max_]
	Number (N)	*X*_1.1_ = 1.10 *X*_mean_	If target magnitude *S*
		*X*_max_ = 1.30 *X*_mean_	*c*_0_ = [*D*_mean_, *N*_mean_]; *c*_min_ = [*D*_min_, *N*_min_]; *c*_max_ = [*D*_max_, *N*_max_]
			If target magnitude *N*
			*c*_0_ = [*S*_mean_, *D*_mean_]; *c*_min_ = [S_min_, D_min_]; *c*_max_ = [*S*_max_, *D*_max_]
	**Instruction**	**Letter stimulus**	**Letter stimulus type**
Target detection task	Red T search	Red upright T	Target
		Red upside-down T	Distractor
		Green upright T	Distractor
		Green upside-down T	Distractor

In the magnitude bisection tasks (top line), one of three dimensions (number, surface, duration) was the target dimension. Four values were tested for the target dimension X (0.7, 0.9, 1.1 and 1.3 * X_mean_), while non-target dimensions Y and Z were determined according to three experimental conditions: in c_0_ non-target dimensions took their middle value (Y_mean_, Z_mean_); in c_min_ non-target dimensions were minimal (Y_min_, Z_min_); in c_max_ non-target dimensions were maximal (Y_max_, Z_max_). In the target detection task (bottom line), participants had to detect a target letter (red upright T) that appeared at each trial within one of the dots and reject the distracters (green upright T, red upside-down T, green upside-down T).

In addition to the magnitude tasks, we also used a target detection control task to measure participants' attention. This aimed at excluding that any generalized impairment in the magnitude tasks could be due to attention-related disorders. For this target detection task a letter appeared inside one of the dots in each trial and could be either a target (red upright T) or a distractor (red inverted T, green upright T or green inverted T).

Trials were pseudo-randomized across tasks and conditions. A total of 720 trials were collected in the magnitude bisection tasks (3 dimensions × 3 conditions × 4 values × 20 trials) and 200 in the control detection task (one third with a target and two thirds with distracters equally presented). Trials were pseudo-randomized across tasks and conditions, and blocked by 100 trials (the original experimental design comprehended two additional conditions, with a total of 1400 trials).

***Procedure***. Before the test session, participants engaged in the training phase: they were familiarized with the minimum (*X*_0.7_) and maximum (*X*_1.3_) values for each magnitude dimension (*D, S, N*) as well as with the target (red upright T) and distracters (green upright T, red or green upside-down T). The training session consisted of two stages: a learning and a test stage. During the learning stage, participants passively viewed 10 examples of stimuli for each task (5 minima and 5 maxima or 4 targets and 6 distracters). They then moved on to the test stage in which they were presented with the same 10 examples and asked to perform a categorical judgment. In the test phase each trial started with one of four instructions: “Duration,” “Surface,” “Number” (magnitude bisection task) or “Red T” (detection task) displayed centrally on the screen for 500 ms. A fixation cross followed for a duration pseudo-randomly chosen between 1500 and 2500 ms after which the stimulus was presented. After the stimulus display and a subsequent 300–500 ms fixation cross, participants were prompted for their response by the simultaneous appearance of “+” and “−” displayed on each side of the fixation cross. In the magnitude tasks (bisection tasks), participants were instructed to judge whether the stimulus displayed was closer to the minimum standard (“−”) or the maximum standard (“+”) in a given dimension. In the control task (target detection) participants were instructed to indicate whether they had seen either the target (“+”) or a distractor (“−”), see Figure 1B. The relative position of “+” and “−” on the monitor was pseudo-randomly assigned throughout the trials. Response keys were “h” and “j” on the computer keyboard. Participants were instructed at the beginning to avoid counting and to respond by hunch. In addition, performance in discriminating durations in this range (700–1300 ms) is unlikely to benefit from using a counting strategy (Grondin et al., 2004). There was no time constraint to respond.

### Data analysis

#### Magnitude bisection tasks

The proportions of “+” responses (stimulus estimated as closer to the maximum standard) were computed separately for each task, dimension and condition. Values were individually fitted to a cumulative Gaussian function *f* using Psignifit 3.0.8 (Fründ et al., 2011) in Matlab 7.0. Two indices were computed: the Point of Subjective Equality (*PSE*, value at 50% of “+” responses) which is a canonical measure of accuracy and the *wf*, computed as in study 1, which reflect sensitivity.

Data were cleaned as follow: when *wf* values were negative or outside ±3 standard deviations of the total mean, data for that participant and in that dimension were excluded (10% of the data were excluded across both groups). In the YA group, 2 participants were excluded from the duration task, 2 from the cumulative area task and 1 from the number task. In the OA group, 2 participants were excluded from the duration task and 2 were excluded from the cumulative area task (resulting in a minimum of 12 participants per dimension in each group).

Separate repeated-measure ANOVAs were performed on PSEs and *wf*s using the IBM SPSS software (Version 19.0). A Greenhouse-Geisser correction was applied when appropriate. *Post-hoc* Bonferroni-corrected *t*-tests were performed to explore significant main effects or interactions.

#### Target detection task

Hit and false alarm rate were computed as the proportion of target which were correctly detected, and the proportion of distractors that were detected as targets, respectively. Dprime (*d′*) detection scores were computed by subtracting the z-scores of hit from false alarm (with *N* the inverse normal law):

$$d^{\prime }=N^{-1}\left(\text{HIT}\right)-N^{-1}\left(FA\right)$$

### Results

#### Background tasks

The two groups differed marginally in the estimate of IQ assessed by the National Adult Reading Test, with OAs slightly outperforming young adults [*t*_(23)_ = −2.03, *p* = 0.054]. Participants in the two groups did not differ in arithmetic performance as measured by the Graded Difficulty Arithmetic test [*t*_(27)_ = −0.065, *p* > 0.9] and the arithmetic subtest of the Wechsler Adult Intelligence Scale-R [*t*_(27)_ = −0.613, *p* > 0.5].

#### Target detection task

Both groups were able to perform the target detection task (*d*'_YA_ = 2.18; *d*'_OA_ = 2.18), with no group difference [independent sample *t*-test on *d*′ values, *t*_(28)_ = 0.02, *p* > 0.9]. This suggests that both groups were equally able to attend to the stimuli throughout the task.

#### Experimental tasks: magnitude bisection tasks

**Figure 3:**
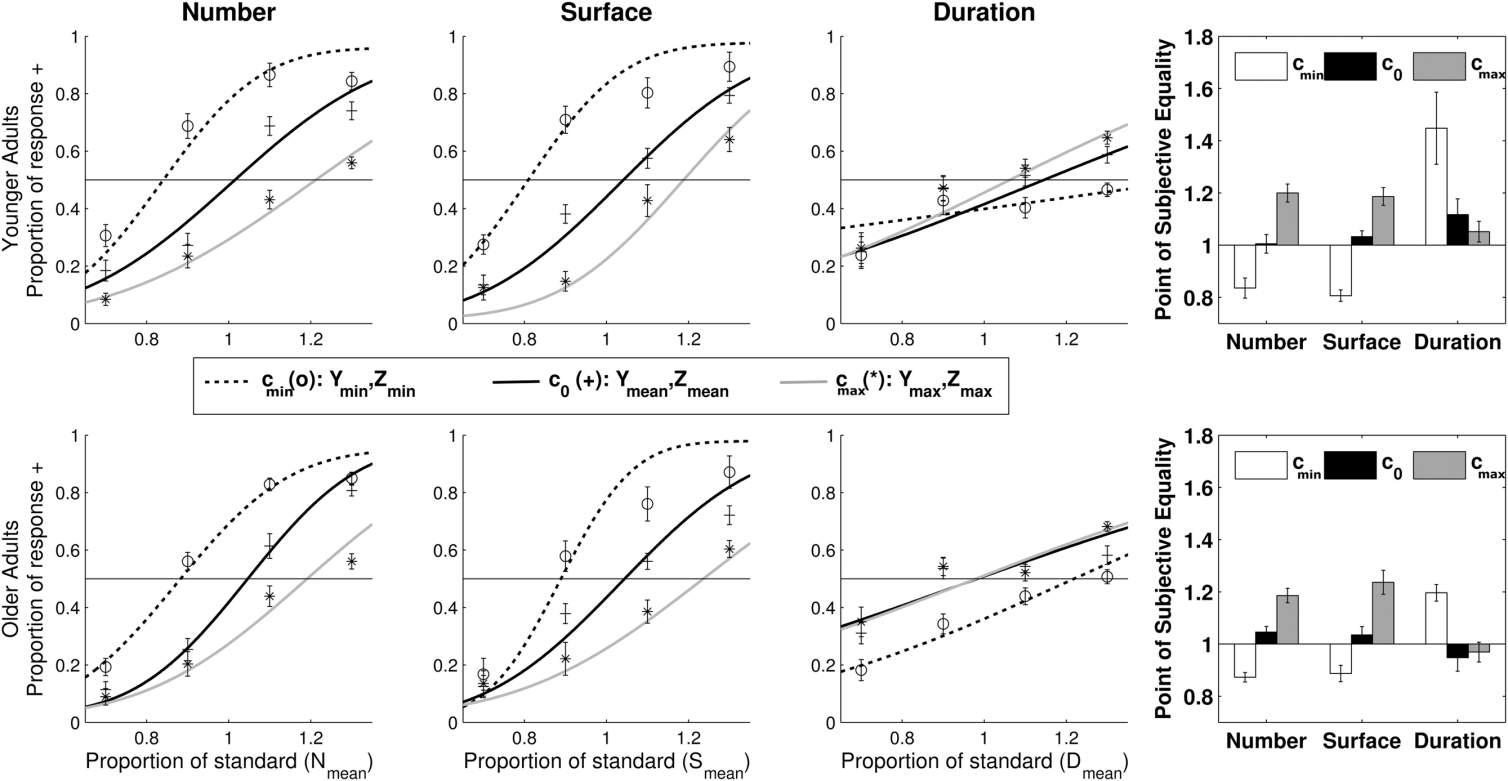
**Performance in the magnitude bisection tasks for the younger adult (Top) and older adult (Bottom) groups**. **Left panel**: psychometric profiles of response for the number, cumulative area and duration tasks. Data points show average responses across participants. Full lines correspond to the average psychometric fit. **Right panel**: Point of Subjective Equality for the number, cumulative area and duration task in the three experimental conditions *c*_0_ (*Y*_mean_, *Z*_mean_), *c*_min_ (*Y*_min_, *Z*_min_) and *c*_max_ (*Y*_max_, *Z*_max_). Error bars show standard error of the mean.

Since our criterion to include or exclude individual participant's data point was applied separately for each task, some participants were retained in one task and not in others. In order to maximize statistical power we therefore conducted statistical analyses on each task separately. Figure 3 shows the psychometric profiles of responses obtained in each group.

***Point of Subjective Equality (PSE)***. Planned mixed-design, repeated-measure ANOVAs with PSE as the dependent variable, condition (3: *c*_0_, *c*_min_, *c*_max_) as independent factor and group (2: YA, OA) as between-group factor were conducted for each task separately. Results are presented in Figure 3 (right panel).

In every task, the ANOVA revealed a significant main effect of condition [number: *F*_(2, 29)_ = 138.64, *p* < 0.001, η^2^_*p*_ = 0.828; cumulative area: *F*_(2, 26)_ = 156.96, *p* < 0.001, η^2^_*p*_ = 0.867; duration: *F*_(2, 26)_ = 11.97, *p* < 0.005, η^2^_*p*_ = 0]. In the *number task*, post-hoc paired-sample *t*-tests revealed that PSE in *c*_min_ was smaller than in *c*_0_ [*t*_(29)_ = 10.93, *p* < 0.001] and PSE in c_0_ was smaller than in *c*_max_ [*t*_(29)_ = −7.22, *p* < 0.001]. Additionally, PSE was smaller in *c*_min_ than in *c*_max_ [*t*_(29)_ = −14.51, *p* < 0.001]. Therefore, number was overestimated when duration and cumulative area were minimal, and underestimated when duration and cumulative area were maximal.

Similarly, in the *cumulative area task*, *post-hoc* paired *t*-tests showed that PSE in *c*_min_ was smaller than in *c*_0_ [*t*_(26)_ = 10.07, *p* < 0.001] and PSE in *c*_0_ was smaller than in *c*_max_[*t*_(26)_ = −9.19, *p* < 0.001]. Additionally, PSE was smaller in *c*_min_ than in *c*_max_ [*t*_(26)_ = −15.15, *p* < 0.001]. Therefore, cumulative area was underestimated when duration and number were maximal, and overestimated when duration and number were minimal.

In the *duration task*, *post-hoc* paired-sample *t*-tests showed that PSE in both *c*_0_ [*t*_(26)_ = −3.57, *p* < 0.005] and *c*_max_ [*t*_(26)_ = 3.70, *p* < 0.005] conditions were smaller than PSE in *c*_min_. Duration was therefore underestimated when cumulative area and number were minimal compared to when they had either mean or maximal values.

Critically, a main effect of group was found in the *duration task* [*F*_(1, 26)_ = 6.70, *p* < 0.05, η^2^_*p*_ = 0.218]. YAs produced a higher PSE than OAs, i.e., OAs overestimated duration relative to YAs (*PSE*_YA_ = 1.206, *PSE*_OA_ = 1.038). This results confirms the idea that temporal estimation changes with age (time seems to stretch for longer). However, the absence of a condition × group interaction indicates that although the absolute perception of duration changes with ageing, the way in which other magnitude dimensions interfere with duration judgment is similar in both age groups.

Overall these results confirm that even when they are task-irrelevant, magnitude dimensions interfere with the accuracy of each other's judgment. In line with previous findings (e.g., Xuan et al., 2007, 2009; Casasanto and Boroditsky, 2008; Oliveri et al., 2008), cumulative area and numerosity affected duration judgment in a positively correlated way (the larger the cumulative area and number, the longer the subjective duration). More surprisingly, duration and number, and duration and cumulative area, affected cumulative area and number judgment in a negatively correlated way, respectively; this means that many dots presented for a longer time appeared to have a small cumulative area, and that larger dots presented for a longer time seemed less numerous. Additionally, while cumulative area and number were perceived similarly by both groups, temporal content was judged shorter in the older than in the YAs group.

***Weber fraction (wf)***. Planned mixed-design, repeated-measure ANOVAs with *wf* as the dependent variable, condition (3: *c*_0_, *c*_min_, *c*_max_) as independent factor and group (2: YA, OA) as between-group factor were run for each task separately. Results are presented in Figure 2 (right panel).

In the *number task*, the ANOVA revealed a main effect of condition [*F*_(2, 29)_ = 10.31, *p* < 0.001, η^2^_*p*_ = 0.276]. Corrected *post-hoc* paired-sample *t*-tests revealed that *wf* was lower in *c*_min_ [*t*_(29)_ = −3.88, *p* < 0.005] and in *c*_0_ [*t*_(29)_ = −2.92, *p* < 0.01] than in *c*_max_. *Wf* was also found to be marginally smaller in *c*_min_ than in *c*_0_ [*t*_(29)_ = 1.94, *p* = 0.062]. This indicates that both groups were less precise to estimate number when space and time had large values than when they had small values. Critically, no main effect or interaction with group was significant, indicating that older and YAs estimated number equally well.

In the *cumulative area task*, the ANOVA revealed a main effect of condition [*F*_(2, 26)_ = 13.10, *p* < 0.001, η^2^_*p*_ = 0.353]. Corrected *post-hoc* paired-sample *t*-tests showed that *wf* was lower in *c*_min_ than both in *c*_0_ [*t*_(26)_ = 6.51, *p* < 0.001] and *c*_max_ [*t*_(26)_ = −3.67, *p* < 0.005], suggesting that both YAs and OAs were more precise to estimate cumulative area when time and number were minimal. Interestingly, the analysis also revealed a significant condition × group interaction [*F*_(2, 26)_ = 4.59, *p* < 0.05, η^2^_*p*_ = 0.161]. *Post-hoc* independent *t*-tests, however, showed no significant difference between YAs and OAs' *wf* in either of the conditions (*c*_0_, *c*_min_, or *c*_max_). Further independent *t*-tests revealed that the interaction was likely driven by the difference between *wf* in *c*_min_ and *c*_max_ (*wf*_c2_ – *wf*_c1_) which differed in YAs and OAs [*t*_(24)_ = −2.51, *p* < 0.05]. Paired *t*-tests indicated that in both groups *wf* were smaller in *c*_min_ than both in *c*_max_ [YA: *t*_(14)_ = −2.84, *p* < 0.05; OA: *t*_(12)_ = −3.29, *p* < 0.01] and c_0_ [YA: *t*_(14)_ = 3.86, *p* < 0.005; OA: *t*_(12)_ = 5.58, *p* < 0.001]. Participants were more precise to judge cumulative area when few dots were presented for a short duration (in *c*_min_) than when many dots were presented for a long duration (in *c*_max_), and even more so in the OAs than in the YAs group. There was no significant main effect of group, confirming that precision in the cumulative area task was very similar in both participants group, although interaction effects were slightly accentuated in the OAs group.

In the *duration task*, the ANOVA revealed a marginal main effect of condition [*F*_(2, 26)_ = 3.37, *p* = 0.076, η^2^_*p*_ = 0.123] and a significant interaction condition × group [*F*_(2, 26)_ = 4.87, *p* < 0.05, η^2^_*p*_ = 0.169]. *Post-hoc* independent *t*-tests revealed that *wf* in *c*_min_ was larger in the YAs than in the OAs group [*t*_(24)_ = 2.01, *p* < 0.05]. Paired *t*-tests further indicated that in the YAs group *wf* was smaller in *c*_max_ and as an index than in *c*_min_ [*t*_(14)_ = 2.33, *p* < 0.05 and *t*_(14)_ = −2.15, *p* = 0.051, respectively] whereas there were no difference between conditions in the OAs group. This indicates that sensitivity for duration increased when cumulative area and number took larger values in the YA group, whereas sensitivity to duration was unaffected by cumulative area and number in the OAs group. There was no significant main effect of group, indicating that overall precision in the duration task was similar in both groups.

Overall our findings indicated that sensitivity to number judgment was modulated by task-irrelevant dimensions similarly in both groups. In contrast, in the cumulative area and duration tasks, the fine pattern of interactions differed between groups. In the cumulative area task, OAs' performance was more sensitive to interaction than that of YAs, whereas in the duration task OAs' performance was more resilient to interaction than that of YAs. However, *wf* did not overall differ between age groups in any of the dimensions, suggesting that overall quantity discrimination is preserved in ageing.

## Discussion

This research evaluated the integrity of quantity processing in healthy ageing. In the first study, we used a two-choice paradigm to investigate continuous quantity processing (space and time discrimination) along with standard measures of cognitive processing. Our results indicate that although elderly participants showed typical age-related decline in memory, attention and executive functions, the ability to judge space and time remained intact. To further assess quantity processing in ageing, we explored the relation between magnitude dimensions whose interactions, so far observed in childhood and in young adulthood, have been taken to suggest the existence of shared or overlapping resources for quantity processing (Gallistel and Gelman, 2000; Walsh, 2003; Bueti and Walsh, 2009; Cantlon, 2012). In a second study, we therefore tested whether processing of number, time and space also interacted with each other in older as well as in younger participants. We found that irrespective of age, number, duration and cumulative area estimations were susceptible to concurrent, task-irrelevant magnitude dimensions, suggesting that quantity processing may be supported by a shared mechanism throughout adulthood. However, the extent to which task-irrelevant dimensions influence the sensitivity of continuous quantity judgments (cumulative area and duration) differed slightly with age. In addition, the percept of duration was found to be modulated by age as elderly adults judged durations close to their veridical values whereas younger adults tended to underestimate duration.

Our results of preserved continuous quantity processing (space and time) in ageing, despite otherwise typical signs of cognitive decline, is to our knowledge the first evidence of the integrity of continuous quantity discrimination in healthy ageing. Together with recent findings showing that numerosity discrimination is also resilient to age (Cappelletti et al., in press), this suggests that non-symbolic quantity processing is generally preserved in healthy ageing. This finding might appear in contrast to other studies showing that flexibility in arithmetical problem solving tasks (e.g., Geary and Lin, 1998; Duverne and Lemaire, 2005; Lemaire and Arnaud, 2008) and performance in temporal estimation tasks (e.g., Block et al., 1998; Baudouin et al., 2006; Lustig and Meck, 2011) decrease with age. However, past research has pointed out that decline in other cognitive functions and processes such as memory, processing speed, attention or executive functions rather than quantity processing itself might account for reduced performance in some numerical and temporal judgment tasks (Salthouse, 1991; Salthouse and Kersten, 1993; Vanneste and Pouthas, 1999; Perbal et al., 2002; Salthouse et al., 2003; Duverne et al., 2008; Cappelletti et al., in press).

Our evidence of maintained quantity processing adds to other cognitive abilities that have proven resilient to ageing, such as verbal memory (vocabulary), implicit memory and emotional processes (Hedden and Gabrieli, 2004), and as such our finding contributes to defining the profile of preserved and declining cognitive abilities in older age (Hedden and Gabrieli, 2004). At present, it is not clear why some cognitive processes are better preserved than others in ageing. One possibility is that quantity-based processes may be more primitive and therefore more robust than later acquired skills such as arithmetic or second-language acquisition. Although quantity processes refine with age, they are in place very early in development (e.g., Xu and Spelke, 2000; Feigenson et al., 2002; Brannon et al., 2007). Their ubiquity makes them crucial to navigate the environment at any age. Preserving them in ageing, either by maintaining the same strategies or by reallocating resources could allow individuals to remain aware of their environment and able to adapt their behavior accordingly.

We also found that OAs showed patterns of interaction among quantities which resemble those observed in children and young adults and which have led to the hypothesis of a common mechanism for time, space and number processing (Walsh, 2003; Bueti and Walsh, 2009; Cantlon, 2012). Although most studies have postulated that interactions result from the automatic mapping of quantities onto a unique mental representation (Henik and Tzelgov, 1982; Dehaene, 1992; Dormal et al., 2006; De Hevia and Spelke, 2010; Chang et al., 2011), others (e.g., Lambrechts et al., in press) proposed that quantity estimates more likely result from Bayesian-like cue-integration whereby the preferred strategy to estimate quantity is to combine cues not only from the target dimension but also from concurrent dimensions. A similar view was expressed in Karolis (2013) and supported by an analysis of the scales for space and number. Here, we found that interactions related to cumulative area and duration (as observed on a measure of sensitivity) were modulated by age. For instance, when judging cumulative area, OAs were more susceptible to task-irrelevant magnitude information than YAs. In contrast, when judging durations, OAs were more resilient to interaction of other magnitude dimensions than YAs. Such observations are difficult to reconcile with the view of aging as a declining evolution. For instance, the Inhibition Deficit theory (Hasher and Zacks, 1988) claims that the ability to inhibit task-irrelevant information decreases with age and would predict that interactions are amplified in ageing. However, this would only account for the group differences obtained in the cumulative area task and not in the duration task. A more parsimonious interpretation would be that the weight with which each dimension affects the others changes with age. The current design and our relatively small sample size in study 2 do not allow us to conclude on this possibility which should be explored in the future using dedicated paradigms.

Interestingly, irrespective of age, the directions of the interactions we observed were different from those often reported in the literature. While space and number positively interacted with time perception (more, larger dots were judged to last longer) similar to previous studies (e.g., Dormal et al., 2006; Xuan et al., 2007, 2009; Casasanto and Boroditsky, 2008; Oliveri et al., 2008; Chang et al., 2011), space and time negatively interacted with number estimates, and number and time negatively interacted with space estimates. For instance, larger dots presented for a longer time were estimated less numerous and more dots presented for a longer time were estimated as covering a smaller space. Previous studies reported the opposite pattern, namely that concurrent quantities positively interact with each other (e.g., Pinel et al., 2004; Dormal and Pesenti, 2007; Javadi and Aichelburg, 2012). These unpredicted results, which replicate recent findings obtained with a similar paradigm (Lambrechts et al., in press), may be explained by differences in the experimental paradigm used here and in past studies. Crucially, in our paradigm information about all three quantity dimensions was designed to accumulate similarly over time to match the intrinsic continuous property of duration. Therefore, participants had to integrate time, space and number over the course of the stimuli presentation and could not access the total cumulative area or total number of dots at any single time point before the end. As a result, the stimulus duration affected the amount of dots presented on the screen at a given time. For instance, given the same number of dots, when the stimulus duration was longer (or shorter), less dots were presented on average at a given moment, which could lead participants to perceive them as less (or more) numerous than veridical, arguably misleading them into underestimating (or overestimating) their number. This contrasts with previous studies in which spatial and numerical information were usually displayed all at once on the screen and stayed for the whole duration of the stimulus presentation (e.g., Xuan et al., 2007, 2009; Oliveri et al., 2008; Chang et al., 2011, but see Casasanto and Boroditsky, 2008). In these studies, participants could estimate space and number as soon as a stimulus was presented, independently from its duration, so time did not impact numerical and spatial processing.

Another unexpected result was that older participants produced smaller PSE than younger participants in the duration task in Study 2 estimates of duration were closest to the veridical value for older than YAs. This finding is in disagreement with past research on time perception in ageing claiming that the ratio of estimated duration on objective duration increases with age, i.e., PSE should be getting larger with age (Block et al., 1998). It should be pointed out that most studies used different paradigms such as duration production and reproduction; importantly they tested longer durations (a few seconds or more) than the ones assessed in the present study. The study by Lustig and Meck (2011) comes closest to the present methodology by using a bisection task with durations ranging from 3–6 s, and reports—similar to previous studies—that OAs produce a larger PSE than YAs. Based on time perception models, differences between YAs and OAs were interpreted by most authors in terms of decreased attentional span in the older participant group, although attentional skills were not directly assessed in these studies. Instead in our study, we controlled for attentional levels which were very similar in both groups. In addition, the use of shorter durations might have attenuated the load on attentional processes to maintain information throughout a trial.

## Conclusion

Here we examined the integrity of continuous quantity processing and the link between number, space and time in ageing. We showed first that discrimination of space and time, much like number, was preserved in ageing. We argued that the resilience of quantity processing skills in ageing may reflect the stability of primitive resources dedicated to quantity processing. Second, extending previous findings obtained with children and young adults, we demonstrated that in older adults, number, space and time interact in discrimination judgments, similar to what is observed in younger participants. However, we found subtle dimension-specific differences in the way concurrent dimensions affected the precision of continuous quantity estimation between younger and older adults which might indicate a change of weight of each dimension within the magnitude processing system.

### Conflict of interest statement

The authors declare that the research was conducted in the absence of any commercial or financial relationships that could be construed as a potential conflict of interest.

## References

[B1] Baddeley A. D. Emslie H. Nimmo-Smith I. (1994). Doors and People: a Test of Visual and Verbal Recall and Recognition. Bury St. Edmunds: Thames Valley Test Company

[B3] Basso G. Nichelli P. Frassinetti F. Di Pellegrino G. (1996). Time perception in a neglected space. Neuroreport 7, 2111–2114 10.1097/00001756-199609020-00009 8930969

[B4] Baudouin A. Vanneste S. Pouthas V. Isingrini M. (2006). Age-related changes in duration reproduction: involvement of working memory processes. Brain Cogn. 62, 17–23 10.1016/j.bandc.2006.03.003 16697513

[B5] Beran M. J. (2007). Rhesus monkeys (*Macaca mulatta*) enumerate large and small sequentially presented sets of items using analog numerical representations. J. Exp. Psychol. Anim. Behave. Process. 33, 42–54 10.1037/0097-7403.33.1.42 17227194

[B6] Birren J. E. Botwinick J. (1955). Speed of response as a function of perceptual difficulty and age. J. Gerontol. 10, 433–436 10.1093/geronj/10.4.433 13263547

[B7] Block R. A. Zakay D. Hancock P. A. (1998). Human aging and duration judgments: a meta-analytic review. Psychol. Aging 13, 584–596 10.1037/0882-7974.13.4.584 9883459

[B7a] Bonn C. D. Cantlon J. F. (2012). The origins and structure of quantitative concepts. Cogn. Neuropsychol. 29, 149–173 10.1080/02643294.2012.707122 22966853 PMC3894054

[B8] Bottini R. Casasanto D. (2010). Implicit spatial length modulates time estimates, but not vice versa, in Processings of the 32nd Annual Conference of the Cognitive Science Society, (Mt. Hood/Portland, OR), 1348–1353

[B9] Brainard D. H. (1997). The psychophysics toolbox. Spat. Vis. 10, 433–436 10.1163/156856897X00357 9176952

[B10] Brannon E. M. Lutz D. Cordes S. (2006). Number representation in infancy. Dev. Sci. 9, 59–64 10.1111/j.1467-7687.2006.00530.x 17059447 PMC1661837

[B11] Brannon E. M. Suanda S. Libertus K. (2007). Temporal discrimination increases in precision over development and parallels the development of numerosity discrimination. Dev. Sci. 10, 770–777 10.1111/j.1467-7687.2007.00635.x 17973794 PMC2918408

[B12] Breukelaar J. W. Dalrymple-Alford J. C. (1998). Timing ability and numerical competence in rats. J. Exp. Psychol. Anim. Behave. Process. 24, 84–97 10.1037/0097-7403.24.1.84 9438968

[B13] Bueti D. Walsh V. (2009). The parietal cortex and the representation of time, space, number and other magnitudes. Phil. Trans. R Soc. B Biol. Sci. 364, 1831–1840 10.1098/rstb.2009.0028 19487186 PMC2685826

[B15] Cantlon J. F. (2012). Math, monkeys, and the developing brain. Proc. Natl. Acad. Sci. U.S.A. 109(Suppl.), 10725–10732 10.1073/pnas.1201893109 22723349 PMC3386867

[B18a] Cappelletti M. Didino D. Stoianov I. Zorzi M. (in press). Number skills are maintained in healthy ageing. Cogn. Psychol. 10.1016/j.cogpsych.2013.11.004 24423632

[B16] Cappelletti M. Freeman E. D. Cipolotti L. (2009). Dissociations and interactions between time, numerosity and space processing. Neuropsychologia 47, 2732–2748 10.1016/j.neuropsychologia.2009.05.024 19501604 PMC2796173

[B17] Cappelletti M. Freeman E. D. Cipolotti L. (2011). Numbers and time doubly dissociate. Neuropsychologia. 49, 3078–3092 10.1016/j.neuropsychologia.2011.07.014 21807010

[B18] Casasanto D. Boroditsky L. (2008). Time in the mind: using space to think about time. Cognition 106, 579–593 10.1016/j.cognition.2007.03.004 17509553

[B19] Casasanto D. Fotakopoulou O. Boroditsky L. (2010). Space and time in the child's mind: evidence for a cross-dimensional asymmetry. Cogn. Sci. 34, 387–405 10.1111/j.1551-6709.2010.01094.x 21564218

[B20] Chang A. Y.-C. Tzeng O. J. L. Hung D. L. Wu D. H. (2011). Big time is not always long: numerical magnitude automatically affects time reproduction. Psychol. Sci. 22, 1567–1573 10.1177/0956797611418837 22042728

[B21] Dehaene S. (1992). Varieties of numerical abilities. Cognition 44, 1–42 10.1016/0010-0277(92)90049-N 1511583

[B21a] De Hevia M.-D. Girelli L. Bricolo E. Vallar G. (2008). The representational space of numerical magnitude: illusions of length. Q. J. Exp. Psychol. 61, 1496–1514 10.1080/17470210701560674 17924288

[B22] De Hevia M. D. Spelke E. S. (2010). Number-space mapping in human infants. Psychol. Sci. 21, 653–660 10.1177/0956797610366091 20483843 PMC3129621

[B23] Doricchi F. Guariglia P. Gasparini M. Tomaiuolo F. (2005). Dissociation between physical and mental number line bisection in right hemisphere brain damage. Nat. Neurosci. 8, 1663–1665 10.1038/nn1563 16261135

[B24] Dormal V. Grade S. Mormont E. Pesenti M. (2012). Dissociation between numerosity and duration processing in ageing and early Parkinson's disease. Neuropsychologia 50, 2365–2370 10.1016/j.neuropsychologia.2012.06.006 22728343

[B23a] Dormal V. Pesenti M. (2007). Numerosity-length interference. Exp. Psychol. 54, 289–297 10.1027/1618-3169.54.4.289 17953149

[B25] Dormal V. Pesenti M. (2013). Processing numerosity, length and duration in a three-dimensional Stroop-like task: towards a gradient of processing automaticity? Psychol. Res. 77, 116–127 10.1007/s00426-012-0414-3 22293902

[B26] Dormal V. Seron X. Pesenti M. (2006). Numerosity-duration interaction: a Stroop experiment. Acta Psychol. 121, 109–124 10.1016/j.actpsy.2005.06.003 16095549

[B27] Droit-volet S. Clément A. Fayol M. (2008). Time, number and length?: similarities and differences in discrimination in adults and children. Exp. Psychol. 61, 1827–1846 10.1080/17470210701743643 19031154

[B30] Droit-Volet S. Clément A. Fayol M. (2003). Time and number discrimination in a bisection task with a sequence of stimuli: a developmental approach. J. Exp. Child Psychol. 84, 63–76 10.1016/S0022-0965(02)00180-7 12553918

[B31] Duverne S. Lemaire P. (2005). Arithmetic split effects reflect strategy selection: an adult age comparative study in addition comparison and verification tasks. Can. J. Exp. Psychol. 59, 262–278 10.1037/h0087479 16459897

[B32] Duverne S. Lemaire P. Vandierendonck A. (2008). Do working-memory executive components mediate the effects of age on strategy selection or on strategy execution. Insights from arithmetic problem solving. Psychol. Res. 72, 27–38 10.1007/s00426-006-0071-5 16838186

[B34] El Yagoubi R. Lemaire P. Besson M. (2005). Effects of ageing on arithmetic problem-solving: an event-related brain potential study. J. Cogn. Neurosci. 17, 37–50 10.1162/0898929052880084 15701238

[B34a] Eriksen B. A. Eriksen C. W. (1974). Effects of noise letters upon the identification of a target letter in a nonsearch task. Percept. Psychophys. 16, 143–149 10.3758/BF03203267

[B35] Fan J. McCandliss B. D. Sommer T. Raz A. Posner M. I. (2002). Testing the efficiency and independence of attentional networks. J. Cogn. Neurosci. 14, 340–347 10.1162/089892902317361886 11970796

[B36] Feigenson L. (2007). The equality of quantity. Trends Cogn. Sci. 11, 185–187 10.1016/j.tics.2007.01.006 17339127

[B37] Feigenson L. Carey S. Spelke E. (2002). Infants' discrimination of number vs. continuous extent. Cogn. Psychol. 44, 33–66 10.1006/cogp.2001.0760 11814309

[B39] Folstein M. F. Folstein S. E. McHugh P. R. (1975). “Mini-mental state.” A practical method for grading the cognitive state of patients for the clinician. J. Psychiatr. Res. 12, 189–198 10.1016/0022-3956(75)90026-6 1202204

[B40] Fründ I. Haenel N. V. Wichmann F. A. (2011). Inference for psychometric functions in the presence of nonstationary behavior. J. Vis. 11, 1–19 10.1167/11.6.16 21606382

[B41] Gallistel C. R. (1989). Animal cognition: the representation of space, time and number. Annu. Rev. Psychol. 40, 155–189 10.1146/annurev.ps.40.020189.001103 2648974

[B42] Gallistel C. R. (2011). Mental magnitudes, in Space, Time and Number in the Brain: Searching for the Foundations of Mathematical Thought, eds Dehaene S. Brannon L. (New York, NY: Elsevier), 3–12 10.1016/B978-0-12-385948-8.00001-3

[B43] Gallistel C. R. Gelman R. (2000). Non-verbal numerical cognition: from reals to integers. Trends Cogn. Sci. 4, 59–65 10.1016/S1364-6613(99)01424-2 10652523

[B44] Gandini D. Lemaire P. Dufau S. (2008). Older and younger adults' strategies in approximate quantification. Acta Psychol. 129, 175–189 10.1016/j.actpsy.2008.05.009 18606394

[B45] Gandini D. Lemaire P. Michel B. F. (2009). Approximate quantification in young, healthy older adults, and Alzheimer patients. Brain Cogn. 70, 53–61 10.1016/j.bandc.2008.12.004 19167145

[B46b] Geary D. C. Lin J. (1998). Numerical cognition: age-related differences in the speed. Exp. Ageing Res. 24, 101 10.1080/036107398244274 9555566

[B46a] Grondin S. Ouellet B. Roussel M.-E. (2004). Benefits and limits of explicit counting for discriminating temporal intervals. Can. J. Exp. Psychol. 58, 1–12 10.1037/h0087436 15072205

[B46] Halberda J. Ly R. Wilmer J. B. Naiman D. Q. Germine L. (2012). Number sense across the lifespan as revealed by a massive Internet-based sample. Proc. Natl. Acad. Sci. U.S.A. 109, 11116–11120 10.1073/pnas.1200196109 22733748 PMC3396479

[B47] Hasher L. Zacks R. T. (1988). Working Memory, comprehension and aging: a review and a new view, in The Psychology of Learning and Motivation, ed Bower G. H. (San Diego, CA: Academic Press), 193–225

[B48] Hedden T. Gabrieli J. D. E. (2004). Insights into the ageing mind: a view from cognitive neuroscience. Nat. Rev. Neurosci. 5, 87–96 10.1038/nrn1323 14735112

[B49] Hellström Å. (1985). The time-order error and its relatives: mirrors of cognitive processes in comparing. Psychol. Bull. 97, 35–61 10.1037/0033-2909.97.1.35

[B50] Helson H. King S. M. (1931). The tau effect: an example of psychological relativity. J. Exp. Psychol. 14, 202–217 10.1037/h007116417799065

[B51] Henik A. Tzelgov J. (1982). Is three greater than five: the relation between physical and semantic size in comparison tasks. Mem. Cogn. 10, 389–395 10.3758/BF03202431 7132716

[B52] Jackson M. Warrington E. K. (1986). Arithmetic skills in patients with unilateral cerebral lesions. Cortex 22, 611–620 10.1016/S0010-9452(86)80020-X 3816245

[B53] Javadi A. H. Aichelburg C. (2012). When time and numerosity interfere: the longer the more, and the more the longer. PLoS ONE 7:e41496 10.1371/journal.pone.0041496 22911801 PMC3401119

[B53a] Javadi A. H. Aichelburg C. (2013). Training enhances the interference of numerosity on duration judgement. PLoS ONE 8:e54098 10.1371/journal.pone.0054098 23326579 PMC3543362

[B54] Karolis V. (2013). The scale analysis of number mapping onto space: manual estimation study. Q. J. Exp. Psychol. 37–41 [Epub ahead of print]. 10.1080/17470218.2013.782325 23590466 PMC4095952

[B55] Kleiner M. Brainard D. Pelli D. (2007). What's new in Psychtoolbox-3? Perception 36, 14 10.1016/0028-3932(83)90075-1

[B55a] Lambrechts A. Walsh V. van Wassenhove (in press). Evidence accumulation in the magnitude system. PlosOne. 10.1371/journal.pone.0082122 PMC3855382 24339998

[B56] Lemaire P. Arnaud L. (2008). Young and older adults' strategies in complex arithmetic. Am. J. Psychol. 121, 1–16 10.2307/20445440 18437798

[B57] Lemaire P. Lecacheur M. (2007). Ageing and numerosity estimation. J. Gerontol. B Psychol. Sci. Soc. Sci. 62, P305–P312 10.1093/geronb/62.6.P305 18079414

[B58] Lourenco S. F. Bonny J. W. Fernandez E. P. Rao S. (2012). Nonsymbolic number and cumulative area representations contribute shared and unique variance to symbolic math competence Proc. Natl. Acad. Sci. U.S.A. 109, 18737–18742 10.1073/pnas.1207212109 23091023 PMC3503215

[B59] Lourenco S. F. Longo M. R. (2010). General magnitude representation in human infants. Psychol. Sci. 21, 873–881 10.1177/0956797610370158 20431048 PMC2930776

[B61] Lustig C. Meck W. H. (2011). Modality differences in timing and temporal memory throughout the lifespan. Brain Cogn. 77, 298–303 10.1016/j.bandc.2011.07.007 21843912

[B62] Masin S. C. Fanton V. (1989). An explanation for the presentation-order effect in the method of constant stimuli. Percep. Psychophys. 46, 483–486 10.3758/BF03210864 2813034

[B63] Meck W. H. (2005). Neuropsychology of timing and time perception. Brain Cogn. 58, 1–8 10.1016/j.bandc.2004.09.004 15878722

[B64] Meck W. H. Church R. M. (1983). A mode control model of counting and timing processes. J. Exp. Psychol. Anim. Behav. Process. 9, 320–334 10.1037/0097-7403.9.3.320 6886634

[B65] Merritt D. J. Casasanto D. Brannon E. M. (2010). Do monkeys think in metaphors? Representations of space and time in monkeys and humans. Cognition 117, 191–202 10.1016/j.cognition.2010.08.011 20846645 PMC2952654

[B66] Nelson H. E. Willison J. R. (1991). National Adult Reading Test (NART). (Windsor, UK: Nfer-Nelson).

[B67] Oliveri M. Vicario C. M. Salerno S. Koch G. Turriziani P. Mangano R. (2008). Perceiving numbers alters time perception. Neurosci. Lett. 438, 308–311 10.1016/j.neulet.2008.04.051 18486340

[B69] Pelli D. G. (1997). The VideoToolbox software for visual psychophysics: transforming numbers into movies. Spat. Vis. 10, 437–442 10.1163/156856897X00366 9176953

[B70] Perbal S. Droit-volet S. Isingrini M. Pouthas V. (2002). Relationships between age-related changes in time estimation and age-related changes. Proc. Speed Attent. Mem. Ageing Neuropsychol. Cogn. 9, 210–216 10.1076/anec.9.3.201.96093428409

[B71] Pinel P. Piazza M. Le Bihan D. Dehaene S. (2004). Distributed and overlapping cerebral representations of number, size, and luminance during comparative judgments. Neuron 41, 983–993 10.1016/S0896-6273(04)00107-2 15046729

[B71a] Posner M. I. Snyder C. R. Davidson B. J. (1980). Attention and the detection of signals. J. Exp. Psychol. 109, 160–174 10.1037//0096-3445.109.2.160 7381367

[B72] Reynvoet B. De Smedt B. Van den Bussche E. (2009). Children's representation of symbolic magnitude: the development of the priming distance effect. J. Exp. Child Psychol. 103, 480–489 10.1016/j.jecp.2009.01.007 19285684

[B73] Roitman J. D. Brannon E. M. Andrews J. R. Platt M. L. (2007). Nonverbal representation of time and number in adults. Acta Psychol. 124, 296–318 10.1016/j.actpsy.2006.03.008 16759623

[B74] Salthouse T. A. (2000). Ageing and measures of processing speed. Biol. Psychol. 54, 35–54 10.1016/S0301-0511(00)00052-1 11035219

[B75] Salthouse T. A. Kersten A. W. (1993). Decomposing adult age differences in symbol arithmetic. Mem. Cogn. 21, 699–710 10.3758/BF03197200 8412720

[B76] Salthouse T. A. (1991). Mediation of adult age differences in cognition by reductions in working memory and speed of processing. Psychol. Sci. 2, 179–183 10.1111/j.1467-9280.1991.tb00127.x

[B77] Salthouse T. A. Atkinson T. M. Berish D. E. (2003). Executive functioning as a potential mediator of age-related cognitive decline in normal adults. J. Exp. Psychol. Gene. 132, 566–594 10.1037/0096-3445.132.4.566 14640849

[B78] Sara M. Faubert J. (2000). Aging, perception, and visual short-term memory for luminance-defined form. Ophthalmic Physiol. Opt. 20, 314–322 10.1016/S0275-5408(99)00095-2 10962697

[B78a] Shuman M. Spelke E. (2006). Object tracking, enumeration, and individuation and element size bias numerosity perception. J. Vis. 6, 777 10.1167/6.6.77716895458

[B79] Stroop J. R. (1935). Studies of interference in serial verbal reactions. J. Exp. Psychol. 18, 643–662 10.1037/h0054651

[B79a] Tokita M. Ishiguchi A. (2011). Temporal information affects the performance of numerosity discrimination: behavioral evidence for a shared system for numerosity and temporal processing. Psychon. Bull. Rev. 18, 550–556 10.3758/s13423-011-0072-2 21390562

[B80] Van Marle K. Wynn K. (2006). Six-month-old infants use analog magnitudes to represent duration. Dev. Sci. 9, F41–F49 10.1111/j.1467-7687.2006.00508.x 16911436

[B81] Vanneste S. Pouthas V. (1999). Timing in ageing?-the role of attention. Exp. Aging Res. 25, 49–68 10.1080/036107399244138 11370109

[B82] Verrillo R. T. (1981). Absolute estimation of line length in three age groups. J. Gerontol. 36, 625–627 10.1093/geronj/36.5.625 7264249

[B82a] Vicario C. M. Pecoraro P. Turriziani P. Koch G. Caltagirone C. Oliveri M. (2008). Relativistic compression and expansion of experiential time in the left and right space. PLoS ONE 3:e1716 10.1371/journal.pone.0001716 18320037 PMC2248621

[B83] Walsh V. (2003). A theory of magnitude: common cortical metrics of time, space and quantity. Trends Cogn. Sci. 7, 483–488 10.1016/j.tics.2003.09.002 14585444

[B84] Wechsler D. (1995). Wechsler Abbreviated Scale of Intelligence. The Psychological Corporation. New York, NY: Harcourt Brace and Company

[B85] Xuan B. Chen X. He S. Zhang D. (2009). Numerical magnitude modulates temporal comparison?: an ERP study. Brain Res. 1269, 135–142 10.1016/j.brainres.2009.03.016 19306851

[B86] Xuan B. Zhang D. Chen X. (2007). Larger stimuli are judged to last longer. J. Vis. 7, 1–5 10.1167/7.10.2 17997671

[B87] Xu F. Spelke E. S. (2000). Large number discrimination in 6-month-old infants. Cognition 74, B1–B11 10.1016/S0010-0277(99)00066-9 10594312

[B88] Zorzi M. Priftis K. Umiltà C. (2002). Neglect disrupts the mental number line. Nature 417, 138–139 10.1038/417138a 12000950

